# Advances in the pharmacological mechanisms of berberine in the treatment of fibrosis

**DOI:** 10.3389/fphar.2024.1455058

**Published:** 2024-09-20

**Authors:** Xiaoqin Liu, Qingzhi Liang, Yifan Wang, Shuai Xiong, Rensong Yue

**Affiliations:** ^1^ Department of Endocrinology, Hospital of Chengdu University of Traditional Chinese Medicine, Chengdu, Sichuan, China; ^2^ Clinical Medical School, Chengdu University of Traditional Chinese Medicine, Chengdu, Sichuan, China; ^3^ Laboratory of Jinfeng, Chongqing, China

**Keywords:** berberine, fibrosis, natural product, pharmacological mechanisms, treatment

## Abstract

The rising incidence of fibrosis poses a major threat to global public health, and the continuous exploration of natural products for the effective treatment of fibrotic diseases is crucial. Berberine (BBR), an isoquinoline alkaloid, is widely used clinically for its anti-inflammatory, anti-tumor and anti-fibrotic pharmacological effects. Until now, researchers have worked to explore the mechanisms of BBR for the treatment of fibrosis, and multiple studies have found that BBR attenuates fibrosis through different pathways such as TGF-β/Smad, AMPK, Nrf2, PPAR-γ, NF-κB, and Notch/snail axis. This review describes the anti-fibrotic mechanism of BBR and its derivatives, and the safety evaluation and toxicity studies of BBR. This provides important therapeutic clues and strategies for exploring new drugs for the treatment of fibrosis. Nevertheless, more studies, especially clinical studies, are still needed. We believe that with the continuous implementation of high-quality studies, significant progress will be made in the treatment of fibrosis.

## 1 Introduction

**FIGURE 1:**
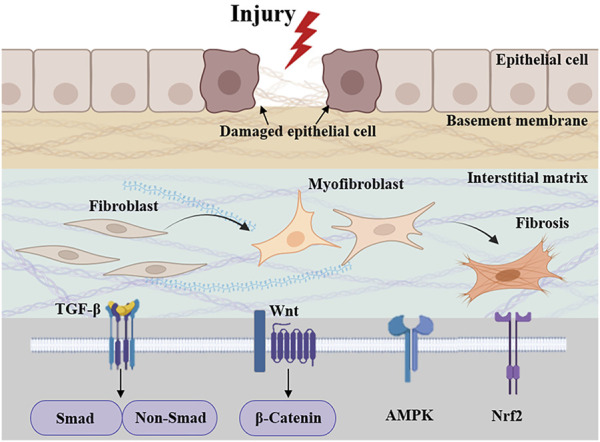
Pathologic processes and signaling cascades associated with fibrosis. Related signaling pathways include TGF-β, AMPK, Nrf2, and inflammatory pathway.

Fibrosis can affect various tissues and organs, with the prevalence of fibrosis-related diseases approaching 5%, and the mortality rate due to fibrosis can be as high as 45% in some regions, resulting in a significant economic burden worldwide (Henderson et al., 2020; Zhao X. et al., 2020). The main pathological changes are excessive deposition of extracellular matrix (ECM), proliferation of fibrous connective tissue and reduction of parenchymal cells in organ tissues, which ultimately leads to structural destruction and functional decline of organs (Friedman et al., 2013; Henderson et al., 2020). Epithelial and endothelial cell damage, inflammatory responses, oxidative stress by various stimuli are common reasons of fibrosis formation and progression (Henderson et al., 2020; Wynn and Ramalingam, 2012; Zhao X. et al., 2020). Common diseases associated with fibrosis include cirrhosis, heart failure, idiopathic pulmonary fibrosis (IPF), nephropathy, diabetes and scleroderma (Zhao X. et al., 2020). For example, hepatic fibrosis (HF) is the result of acute or chronic liver injury, but the result of progressive ECM deposition can lead to histologic cirrhosis (Saxena and Anania, 2015; Zhao X. et al., 2020). Reactive and progressive interstitial fibrosis caused by persistent activation of myocardial fibroblasts leads to myocardial stiffness and ultimately to ventricular dysfunction (Wynn and Ramalingam, 2012). Multiple gene regulatory pathways are critical for driving the function of MFBs, including molecules downstream of the TGF-β receptor, including Smads (Inagaki and Okazaki, 2007; Zhao X. et al., 2020), and extracellular signaling regulators of AMP-activated protein kinase (AMPK), nuclear factor-erythroid 2-related factor 2 (Nrf2), and the inflammatory pathway (Zhao X. et al., 2020), which normally control fibrotic cell motility, proliferation and morphology through well-characterized intracellular signaling pathways (Casaletto and McClatchey, 2012) (Figure 1). Although a small number of drugs are available for the treatment of fibrosis, such as Nidanib and Pirfenidone, long drug cycles, adverse effects, and the lack of drugs targeting different organs remain a challenge to clinical treatment. Therefore, effective therapeutic strategies for fibrosis are urgently required.

Natural products offer clear advantages in the treatment of diseases due to their high safety profile and diverse efficacy (Liu et al., 2024; Wang et al., 2024a). *Rhizoma Coptidis* is well known traditional Chinese medicine used in the treatment of fibrosis, inflammation, diabetes, etc. (Ma et al., 2024; Xiao et al., 2021; Yang et al., 2024). *Rhizoma Coptidis* was first described in *Shennong Bencao Jing* (Tan et al., 2016), and its anti-diabetic properties were first documented in *Note of Elite Physicians* (Zhang Q. et al., 2014). Berberine (BBR), one of the representative active ingredients of *Rhizoma Coptidis* (Shao et al., 2021), is an isoquinoline alkaloid and is also found in the leaves, twigs, barks, rhizomes, roots and stems of large numbers of plants, including Berberis kansuensis C.K. Schneid. (Berberidaceae), Poppyaceae, Coscinium fenestratum (Goetgh.) Colebr. (Menispermaceae), Phellodendron amurense Rupr. (Rutaceae), and Argemone mexicana L. (Papaveraceae) (Feng X. et al., 2019; Habtemariam, 2020; Imenshahidi and Hosseinzadeh, 2016; Singh and Mahajan 2013; Sun et al., 2023; Xu X. et al., 2021). It is an over-the-counter drug for the treatment of bacterial diarrhea and has a long history of medicinal use in both traditional Chinese medicines (Dang et al., 2020; Kong et al., 2020). Multiple studies have demonstrated that BBR has anti-inflammatory, anti-bacterial, anti-viral and anti-fibrotic, and has attracted intensive interest in the treatment of fibrosis of liver, heart, lung, kidney, pancreatic and other organs (Bansod et al., 2020; Che et al., 2019; Chitra et al., 2013; Gao et al., 2024; Tan E. et al., 2023; Xu X. et al., 2021; Zhang Z. et al., 2016).

Literature searches were performed using PubMed, Web of Science, and Google Scholar databases, and no filters were set for the searches in these three databases. The keywords included “berberine”, “fibrosis”, “anti-fibrotic effect”, “pharmacological effect”, “pharmacological mechanism”, “pharmacokinetics”, “safety” and “toxicity”. All articles included were published between 2004 and 2024. The purpose of this review is to focus on the therapeutic role and mechanism studies of BBR in fibrosis, and for this purpose, we read the titles, abstracts, and full text of publications from the past 20 years, removed publications that were not relevant to the topic of this review, and categorized them according to the type of fibrosis and mechanism. We also performed a correlation search for citations to relevant studies and review literature. Duplicates were removed using Endnote 20’s automatic and manual duplicate detection system, and a total of 168 references were finally included.

## 2 Anti-fibrotic effect of BBR

### 2.1 Hepatic fibrosis

**FIGURE 2:**
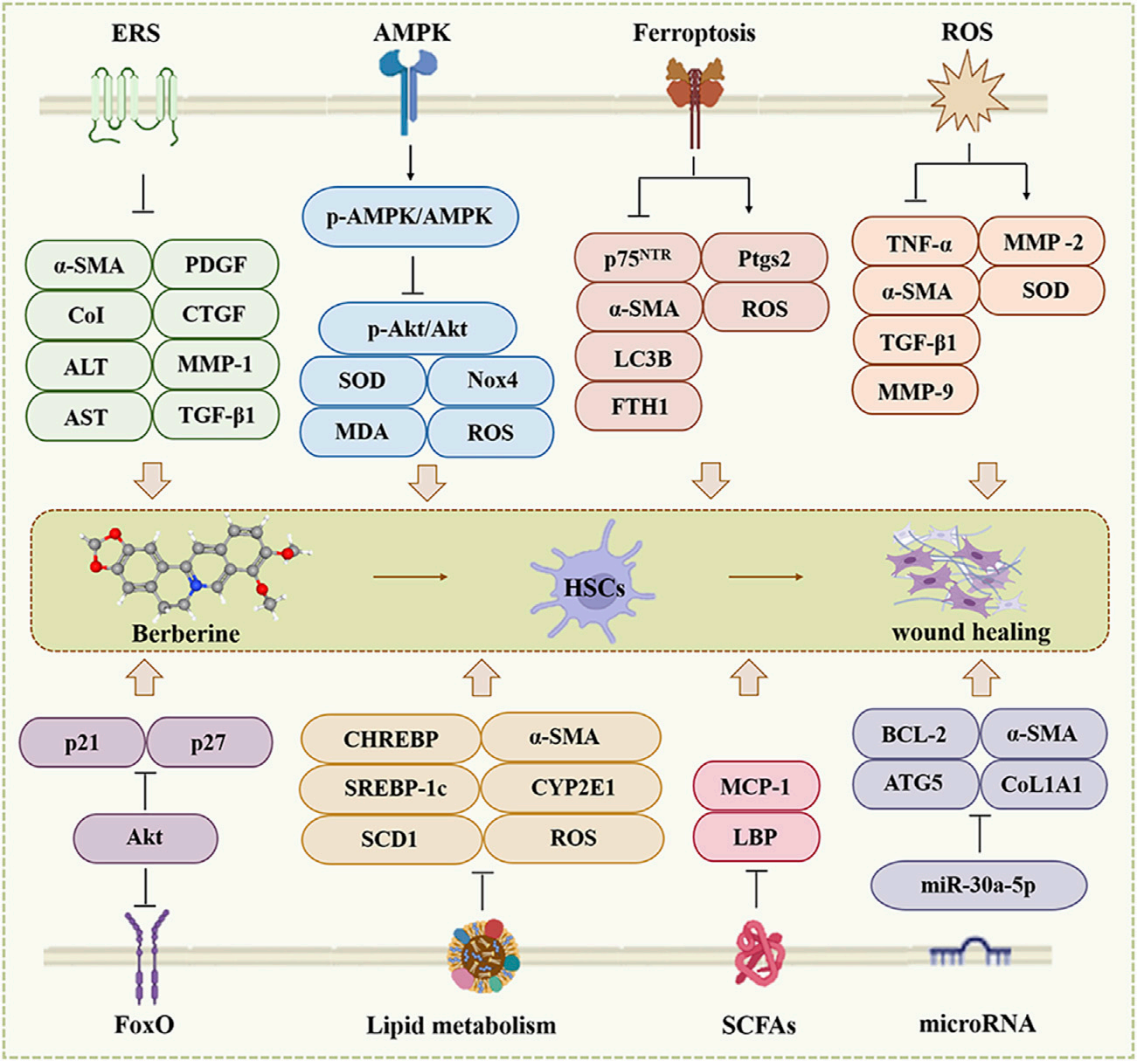
The effect of BBR on the hepatic fibrosis. ERS, AMPK, Ferroptosis, ROS, FoxO, Lipid metabolism, SCFAs and microRNA are involved in BBR for hepatic fibrosis.

The main pathological features of HF are activation of hepatic stellate cells (HSCs) and excessive deposition of protofibrillar collagen (Tacke and Weiskirchen, 2012; Yi et al., 2021). After liver injury, multiple factors such as cytokines, chemokines or reactive oxygen species (ROS) induce the differentiation of HSCs from quiescent to myofibroblasts (MFBs), and the overproduction of smooth muscle α-actinin (α-SMA), which ultimately leads to the overproduction and deposition of ECM components and the development of HF (Kong et al., 2012; Sanchez-Valle et al., 2012; Zimmermann and Tacke, 2011). BBR inhibits the activation of HSCs and the generation of α-SMA and suppresses the development of HF through multiple mechanisms (Figure 2).

#### 2.1.1 Endoplasmic reticulum stress

Fibrotic signaling triggers transcription of procollagen I, which enters the endoplasmic reticulum and is released into the ECM via the endoplasmic reticulum (ER)-Golgi secretion compartment (Ajoolabady et al., 2023; Maiers et al., 2017). Perturbation of ER export of procollagen I induced ER stress (ERS) and UPR activation, leading to apoptosis in HSCs and upregulates fibrotic genes and Smad2 expression, accelerated progression of HF (Koo et al., 2016; Maiers et al., 2017; Xuan et al., 2021).

Previous studies clarified that 200 mg/kg of BBR for 5 weeks downregulated the expression of α-SMA, CoI, alanine aminotransferase (ALT), azelaic transaminase (AST), platelet-derived growth factor (PDGF), connective tissue growth factor (CTGF), metalloproteinase-1 (MMP-1), and TGF-β1 *via* ERS, and attenuated chronic liver injury, inflammation and fibrosis *in vivo* (Zhang Z. et al., 2016). On the other hand, BBR attenuated tunicamycin-induced triglyceride (TG) and collagen deposition in the liver of mice, compared to the tunicamycin group, BBR (75, 150 and 300 mg/kg) all reversed the levels of unfolded protein response (UPR)-related genes (*CHOP*, *GRP78* and *ATF6*) that it upregulated, mainly by attenuating ERS (Yang et al., 2022). In summary, BBR can improve HF by modulating ERS.

#### 2.1.2 AMPK pathway

AMPK, acts as a “metabolic sensor”, to regulate energy homeostasis and metabolic processes (Hardie et al., 2012; Li et al., 2014; Zhao P. et al., 2020). Researchers find AMPK regulates the activation of HSCs and inhibits TGF-β-induced fibrotic properties of HSCs, ameliorating liver injury and fibrosis (da Silva Morais et al., 2009; Zhao P. et al., 2020).

BBR (50 mg/kg) attenuated carbon tetrachloride (CCl4)-induced hepatic histological changes in mice, such as hepatocyte necrosis, adjacent hepatocyte steatosis, ballooning degeneration, lymphocyte infiltration, pseudofollicular and bridging formation through up-regulating the p-AMPK/AMPK ratio in activated HSCs, thereby decreasing the ratio of p-Akt to total Akt, strongly increased serum and liver tissue superoxidedismutase (SOD) activity in CCl4-induced hepatic injury in mice, decreased serum Malondialdehyde (MDA) level and NADPH oxidase 4 (NOX4) expression in liver tissues, reducing ROS production and effectively preventing HF (Li et al., 2014).

#### 2.1.3 Ferroptosis

Emerging research suggests that ferroptosis is characterized by redox-active iron accumulation, ROS generation, lipid peroxidation, and glutathione depletion, and the iron death inhibitor fer1 helps to inhibit the activation of HSCs *in vitro* (Yi et al., 2021).

In thioacetamide (TAA) and CCl4 induced HF in mice, compared to the model group, another proposed mechanism of BBR associated anti-fibrotic effect was based on inhibits HSCs activation through ferroptosis, by which BBR reduced expression of hyaluronic acid (HA), p75^NTR^ (HSC activation marker), ALT, AST and iron deposition, as well as lowering of Ishak score (Passino et al., 2007; Yi et al., 2021). Furthermore, treatment with 21 µM BBR for 24 h affected HSCs but not hepatocytes and reduced TAA-induced α-SMA expression, modulating the viability and proliferation of HSCs in a dose- and time-dependent manner (Yi et al., 2021). Besides, studies have shown that BBR treatment can decreased LC3B and FTH1 expression, enhanced Ptgs2 and ROS levels, promoted ferritin hydrolysis, and increased iron overload in HSCs, suggesting that BBR enhances HSCs ferroptosis through inhibition of autophagy, which is beneficial to HF (Yi et al., 2021). In addition, BBR inhibited the proliferation of HSCs in a dose-dependent manner and its IC_50_ value is 66.86 mM, but combination with sorafenib (10 µM) reduced it to 15.61 µM; molecular docking experiments further demonstrated that BBR binds to PEBP1 (can trigger ferroptosis), with a maximum binding energy of −8.51 kcal/mol; so BBR can promote HSC ferroptosis to alleviate HF through binding to PEBP1 (Xie et al., 2023).

#### 2.1.4 Oxidative stress

Oxidative stress is a vital factor in the pathogenesis of HF. Injured hepatocytes release ROS and inflammatory cytokines which are involved in HSCs activation and recruitment of immune cells to liver tissue (Che et al., 2023; Li N. et al., 2021; Xu et al., 2022). Among them, interleukin 17 (IL-17) promotes ECM production by HSCs and exacerbates HF (Ma et al., 2020; Meng et al., 2012; Seo et al., 2016). Thus, hepatic ROS trigger complex interactions between activated HSCs and recruited immune cells, thereby exacerbating fibrosis and inflammation within the liver.

Accumulating evidences demonstrated that BBR hinder HF through oxidative stress. In the CCL4-induced HF model in Balb/c mice, different doses of BBR (9 and 50 mg/kg) were treated for 2 weeks that ameliorated the increase in plasma enzyme activities and oxidative stress, decreased tumor necrosis factor-α (TNF-α), α-SMA, transforming growth factor beta 1 (TGF-β1) and MMP-9 expression, the levels of MMP-2 is increased, and induced the Cu/Zn SOD activity to be normalization (Domitrovic et al., 2013b). BBR reduced ALT, AST, MDA and hydroxyproline (HYP) levels, as well as increased SOD levels, compared with the CCL4-induced fibrosis group of male Wistar rats (Zhang et al., 2008). BBR (5 µM) can reduced cellular steatosis, hindered ROS, inflammatory cytokines and collagen production *in vitro* (Rafiei et al., 2023). This indicate that BBR treatment can promote liver repair by ameliorating oxidative stress.

#### 2.1.5 Metabolism

Disorders of lipid metabolism, such as fatty acid and cholesterol metabolism, are one of the pathologic bases of many liver diseases (Arain et al., 2017; Huang et al., 2021; Liu et al., 2022; Scorletti and Carr, 2022). In non-alcoholic fatty liver disease, dysregulation of the lipid balance in the body can lead to a severe accumulation of triacylglycerols in the liver cells, which can progress to non-alcoholic steatohepatitis, hepatic fibrosis and even cirrhosis (Grabner et al., 2021). Lipid metabolism in the liver is regulated by various mechanisms, including peroxisome proliferator-activated receptors (PPARs) and sterol regulatory element binding protein (SREBP) (Grabner et al., 2021).

BBR downregulates the expression of the lipid metabolism-related gene stearoyl coenzyme A desaturase 1 (SCD1) (Yang et al., 2022), reduces TG biosynthesis and enhances TG oxidation to ameliorate hepatic steatosis and HF *in vivo* (Bansod et al., 2021; Boudaba et al., 2018; Huang et al., 2021; Wang et al., 2016; Zhang et al., 2019). Other studies have reported that BBR supplementation improves total cholesterol, low-density lipoprotein C and high-density lipoprotein C in the blood and accelerates cholesterol excretion through inhibition of adipocyte enhancer-binding protein one or enhancement of cholesterol-binding receptor, which explains its hepatoprotective properties *in vivo* and *in vitro* (Derosa et al., 2013; Kong et al., 2004; Wang and Zidichouski, 2018; Wei et al., 2016).

PPARs are considered important metabolic regulators of hepatic lipid metabolism and inflammation (Boyer-Diaz et al., 2021; Chen et al., 2023). PPAR-γ inhibits the activation of HSCs and regulates the expression of genes related to adipogenesis and fibrogenesis to prevent HF progression (Brunner et al., 2019; Perez-Carreon et al., 2010; Sanyal et al., 2010; Yuan et al., 2004). BBR enhances the expression of the gene encoding the FAO carnitine palmitoyl transferase IA by interacting with PPAR-α to inhibit the production of TG *in vivo* (Yu et al., 2016; Zhou et al., 2008). BBR contains potential agonists of all PPAR isoforms (Xia et al., 2013; Yu et al., 2016), and these could act as ligands to regulate the progression of HF. Taken together, BBR may restore lipid homeostasis and regulate liver function by modulating the PPARs signaling cascade.

SREBP-1c, CHREBP, FAs and C/EBPβ as adipogenic regulators, are involved in the regulation of steatosis, inflammation and fibrosis, however, BBR downregulates their mRNA levels in tunicamycin-induced mice, and mRNA levels of ROS, CYP2E1 (a major mediator of lipid peroxidation), and the expression of TNF-α, IL-6, α-SMA, TIMP-1 and TGF-β1 that are the anti-fibrotic effect of BBR was fully utilized (Zhang Z. et al., 2016).

#### 2.1.6 Gut microbiota

Gut microbiota is a complex system that regulates certain biochemical, physiological and immune responses to maintain the health of the organism (Mishra et al., 2021). It alters the microenvironment of the organism by directly influencing metabolic signaling and energy metabolism either by itself or by producing certain metabolites, which can lead to inflammation, autoimmunity and metabolic disorders (Thursby and Juge, 2017). Thus, a growing body of research suggests that ecological dysregulation of the gut microbiota and impairment of its composition and function are strongly associated with many metabolic diseases (Cai and Kang, 2023; Wang et al., 2021).

Short-chain fatty acids (SCFAs), the end products of anaerobic microbial fermentation of indigestible carbohydrates, have a profound impact on gut function and host energy metabolism (Nicholson et al., 2012). BBR (100 mg/kg) treatment increased the concentration of SCFAs in the gut, increased the production of gut flora-derived butyrate, and decreased lipopolysaccharide (LPS) binding protein (LBP), monocyte chemotactic protein-1 (MCP-1), leptin and lipocalin, and promotes homeostasis of the hepatic microenvironment *in vivo* (Zhang et al., 2012).

#### 2.1.7 Forkhead box O pathway

Forkhead box O (FoxO) is an essential class of transcription factors involved in numerous biological processes, such as cell cycle, cell proliferation, apoptosis and anti-oxidative stress (Calissi et al., 2021; Sun et al., 2009). p21 and p27 are FoxO-specific transcriptional targets that are associated with G1-phase cell cycle arrest (Roy et al., 2011; Sun et al., 2009). p27 is the key downstream target of FoxO1 to control proliferation and differentiation of HSCs (Sun et al., 2009). The protein kinase B (Akt) signaling cascade controls the transcriptional activity of p21 and p27 through phosphorylating FoxO1 reversing its transcriptional activity (Sun et al., 2009).

This study reported that BBR (5, 10 and 20 μg/mL) induced subcellular redistribution of FoxO1 from the cytoplasm to the nucleus in hepatic stellate cells (CFSCs) *in vitro*, which reduced the number of activated HSCs, fibrotic septa, and hepatic collagen. Furthermore, it decreases p-FoxO1 (Ser-256) and p-Akt, increases p21 and p27 expression, and inducts G1 blockade, which directly inhibited the CFSC proliferation, which has a protective effect on the liver (Sun et al., 2009).

#### 2.1.8 Another pathway

Numerous studies have proved that enhanced autophagy can induce fibrotic diseases in many organs (Marcelin et al., 2020; Shang et al., 2020; Zhang et al., 2020). After liver injury, increased autophagy promotes the activation of HSCs (Thoen et al., 2011; Wang B. et al., 2017; Zhang et al., 2017). Different microRNAs (miR) involved in various fibrotic diseases. MiR-15 family mainly promotes cell proliferation and apoptosis. MiR-199 and miR-200 families are responsible for ECM deposition and pro-fibrotic cytokine release (Jiang et al., 2017; Schmiedel et al., 2015).

BBR reduced the expression of ATG5, BCL-2, HYP, α-SMA, collagen type I alpha (COL1A1), LC3, ALT, and AST, attenuated collagen deposition and inflammatory cell infiltration, and up-regulating the expression of p53, BAX, and cleaved PARP, and enhanced hepatic repair through up-regulating the expression of miR-30a-5p *in vivo* and *in vitro* (Tan Y. et al., 2023). Cyclooxygenase-2 (COX-2) is rapidly synthesized by cells in response to various stimuli involving different pathophysiological processes. BBR can inhibit the expression of COX-2 *in vivo* and *in vitro* to protect cells from excessive inflammatory responses, which is beneficial to HF (Feng et al., 2012; Guo et al., 2008; Li et al., 2012; Zeng et al., 2011).

Overall, BBR can mediate different signaling cascades such as ERS, AMPK, ferroptosis, oxidative stress, metabolism, gut microbiota, and FoxO to exert anti-fibrotic effects (Figure 2). However, HF is a complex process, and the activation of HSCs and excessive deposition of collagen fibers are necessary processes that must be taken into account in the future development of new target mechanisms and drugs.

### 2.2 Myocardial fibrosis

**FIGURE 3:**
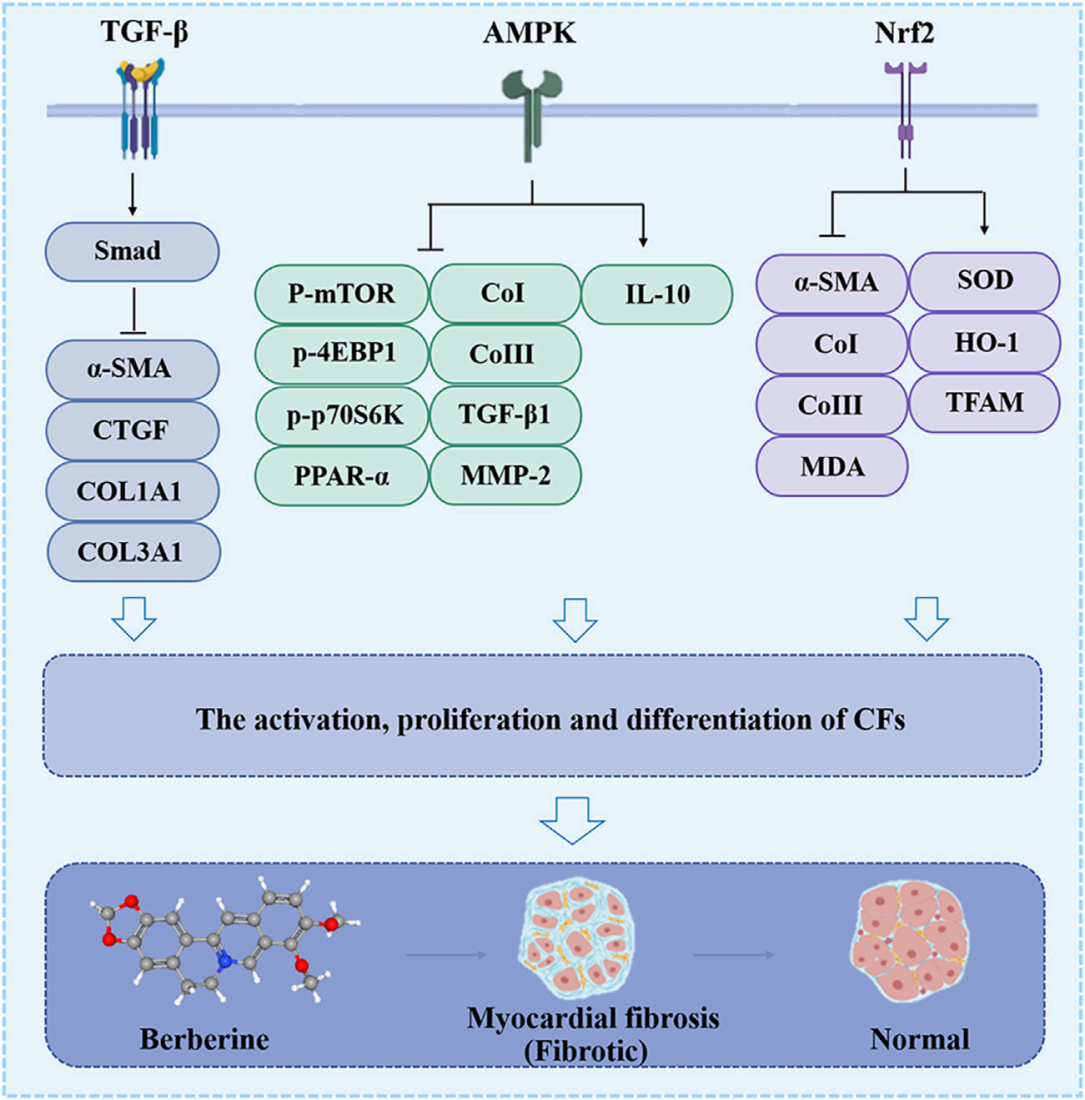
The effect of BBR on the myocardial fibrosis. Related signaling pathways include TGF-β, AMPK and Nrf2 pathway.

One of the major events that occurs in myocardial fibrosis (MF) when the heart is injured is the activation and differentiation of cardiac fibroblasts (CFs) into MFBs, which contribute to ECM turnover and collagen deposition (Liu et al., 2021). Activated CFs are central cellular effectors of MF and are the main source of ECM proteins in MF (Frangogiannis, 2021). BBR hinders CFs activation and differentiation, and reduces ECM production to achieve anti-MF (Figure 3).

#### 2.2.1 TGF-β/smad pathway

Previous studies elaborated that TGF-β producing inflammatory cells are essential in the critical cellular event of CFs activation and ECM deposition (Frangogiannis, 2020; Liu et al., 2021). In isoprenaline (ISO)-induced MF in Sprague-Dawley rats, BBR (10, 30 and 60 mg/kg) attenuated macrophage infiltration, altered macrophage phenotype, reduced the expression of COL1A1, collagen type III alpha (COL3A1), CTGF, TGF-β1, and α-SMA, and inhibited the proliferation of CFs, all of which were achieved by blocking the activation of the TGF-β1/Smad signaling cascade (Che et al., 2019).

#### 2.2.2 AMPK pathway

Several studies have confirmed that BBR downregulates the p-mTOR, p-4EBP1 and p-p70S6K (Thr389), inhibits the proliferation of CFs and their conversion to MFBs, reduces the expression of CoI, CoIII, TGF-β1, MMP-2 and MMP-9, shrinks the size of the MFs, increases the secretion of IL-10, and ultimately inhibits MFs and improves cardiac dysfunction *in vivo* and *in vitro*. The pharmacological mechanism of the above results is strongly related to the phosphorylation of AMPK (Ai et al., 2015; Chen et al., 2020).

#### 2.2.3 Nuclear factor-erythroid 2-related factor 2 pathway

Nuclear factor-erythroid 2-related factor 2 (Nrf2) translocate into the nucleus as a transcription factor and induces the expression of downstream anti-oxidant and detoxification enzymes, such as heme oxidase-1 (HO-1), SOD, Glutathione peroxidase (GPx) (Wang et al., 2023). Nrf2 can counterbalances oxidative stress, also affects TGF-β1-mediated phosphorylation of Smad3, which is indispensable for fibrosis development (Kobayashi et al., 2016).

Recent studies have elucidated that in the DOX-induced MF model in male Sprague-Dawley rats, BBR (60 mg/kg) targeting Nrf2 can downregulated the expression of α-SMA, CoI, CoIII, and MDA, inhibited the differentiation of CFs to MFBs, and increased SOD activity, HO-1, and mitochondrial transcription factor A (TFAM), rescuing mitochondrial morphology damage and loss of membrane potential (Wang et al., 2023), further protecting the normal functioning of the heart.

Taken together, the conversion of CFs to MFBs occurs at a central event in CF, and BBR prevents this event through the TGF-β/Smad, AMPK, and Nrf2 pathways (Figure 3). Hitherto, there is still a critical lack of research on the target and mechanism of BBR in MF treatment.

### 2.3 Pulmonary fibrosis

**FIGURE 4:**
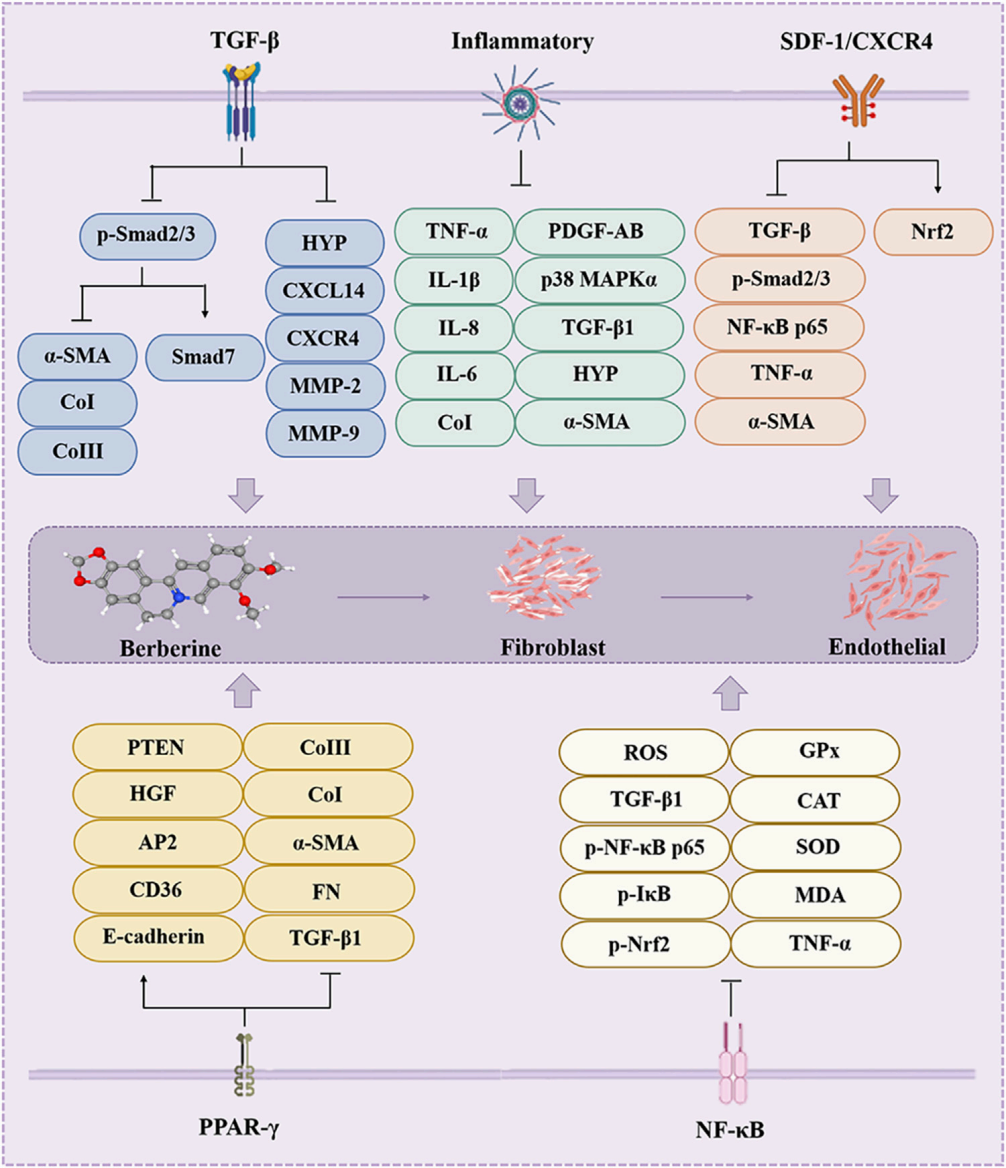
The effect of BBR on the pulmonary fibrosis. Inflammatory, AMPK, TGF-β, Nrf2, Notch/Snail and SIRT2 are involved in BBR for pulmonary fibrosis.

Pulmonary fibrosis (PF) is a heterogeneous lung mesenchymal disorder that includes persistent epithelial injury, abnormal wound healing, and excessive ECM deposition (Dong and Ma, 2016). MFBs, fibroblasts, and other cell types that secrete numerous of ECM into the alveolar structures, continued differentiation, proliferation, and migration of fibroblasts triggered by a variety of fibrotic mediators contribute to the formation of fibrotic foci and subsequent lung structural remodeling (Wang et al., 2024b). However, BBR plays a unique role in the treatment of PF (Figure 4).

#### 2.3.1 TGF-β pathway

TGF-β, a critical inducer of epithelial-mesenchymal transition (EMT), promotes EMT in alveolar epithelial and endothelial cells, restores tissue morphology and structure, and induces fibroblasts to convert to MFBs, synthesize and secrete ECM (Li et al., 2011; Tew et al., 2020). TGF-β-mediated Smad and non-Smad signaling cascades are thought to be major players in accelerated PF(Higashiyama et al., 2007; Wynn, 2011). In the BLM-induced PF in male Wistar albino rats, BBR (200 mg/kg) reverses bleomycin (BLM)-induced lung ultrastructural changes, enhances Smad7 expression, and downregulates α-SMA, CoI and CoIII expression through inhibits BLM-induced elevation of p-Smad 2/3 (Chitra et al., 2015; Tan E. et al., 2023; Palanivel et al., 2015). Another study clarified that BBR reduced the levels of HYP, CXCL14, CXCR4, CoI/III, MMP-2, MMP-9, α-SMA and p-Smad 2/3, and prevented the activation of Smad2/3, ensured recovery of lung status and function (Li et al., 2019).

#### 2.3.2 Inflammatory

Pathologically, PF is always first accompanied by an inflammatory response (Feng F. et al., 2019), inflammatory cells multitask at the wound site by facilitating wound debridement and producing chemokines and growth factors (Eming et al., 2017).

In the PM2.5-induced MF in male C57BL/6 mice and BLM-induecd MF in male Kunming mice, BBR (50 mg/kg) treatment reduces inflammatory cell aggregation in the lungs and decreases the expression of pro-inflammatory cytokines TNF-α, IL-8, IL-1β, and IL-6; it also downregulates the levels of COI, COIII, TGF-β1, PDGF-AB, HYP, α-SMA, p38 MAPKα, and p38 MAPKα (pT180/Y182) and inhibits collagen production and deposition (Alnuqaydan et al., 2022; Beik et al., 2023; Tew et al., 2020; Yang et al., 2023; Zhao et al., 2023).

#### 2.3.3 Stromal cell-derived factor-1/CXCR4 pathway

CXCL12 is involved in the mobilization of bone marrow-derived stem cells through Stromal cell-derived factor-1 (SDF-1) receptor C-X-C chemokine receptor 4 (CXCR4), which is abundantly expressed on a wide range of cells and may be considered a permanent reservoir for fibroblasts (Ahmedy et al., 2023).

Recently, a study found that in the BLM-induecd MF in albino male mice, BBR (5 mg/kg) greatly inhibited BLM-induced weight loss and elevated lung index, preserved lung structure and attenuated bronchoalveolar injury, and ameliorated lung injury, which through reduced expression of SDF1 and CXCR4 (Ahmedy et al., 2023). And further inhibited the expression of TGF-β, p-Smad2/3, α-SMA, NF-κB p65, TNF-α, IL-6, MDA, GSH, SOD and CAT, effectively attenuated oxidative stress, enhanced the expression of Nrf2, and ultimately attenuated epithelial mesenchymal transition of PF (Ahmedy et al., 2023).

#### 2.3.4 PPAR-γ pathway

BBR can directly enter the cytoplasm of fibroblasts and act as a PPAR-γ agonist, up-regulating the nuclear translocation, DNA-binding activity, and transcriptional activity of PPAR-γ, and reversing PM2.5-induced collagen deposition, the expression of fibroblast markers (TGF-β1, FN, α-SMA, COI, and COIII), and the expression of E cadherin expression upregulation, promotes CD36 and AP2 mRNA expression, HGF and PTEN protein levels, and attenuates oxidative and inflammatory factor-mediated PF in BLM-induecd female ICR mice (Guan et al., 2018; Zhao et al., 2023).

#### 2.3.5 Nuclear factor-κB pathway

The BBR-mediated nuclear factor-κB (NF-κB) pathway attenuated the extent of lung oxidative damage and PF. In BLM-induecd male wistar albino rats, BBR (200 mg/kg) action was manifested as inhibition of TGF-β1, ROS, iNOS, TNF-α, MDA, OH, NO, and myeloperoxidase (MPO) levels through downregulation of the phosphorylation of Nrf2, IκB, and NF-κB p65; inhibition of HYP and histamine release, significant reduction of mast cell recruitment, and reversal of BLM-induced SOD, CAT, GPx and GR activities, and restored the levels of non-enzymatic antioxidant status of glutathione, vitamin A, vitamin C and vitamin E (Chitra et al., 2013; Sun et al., 2022).

The special structure of the lung poses a challenge for the treatment of PF, and although BBR can protect the lung structure and reduce lung damage through various pathways (such as TGF-β) (Figure 4), researchers are encouraged to conduct in-depth studies, especially clinical studies, to provide stronger and more comprehensive evidence for the treatment of fibrotic diseases with BBR.

### 2.4 Renal fibrosis

**FIGURE 5:**
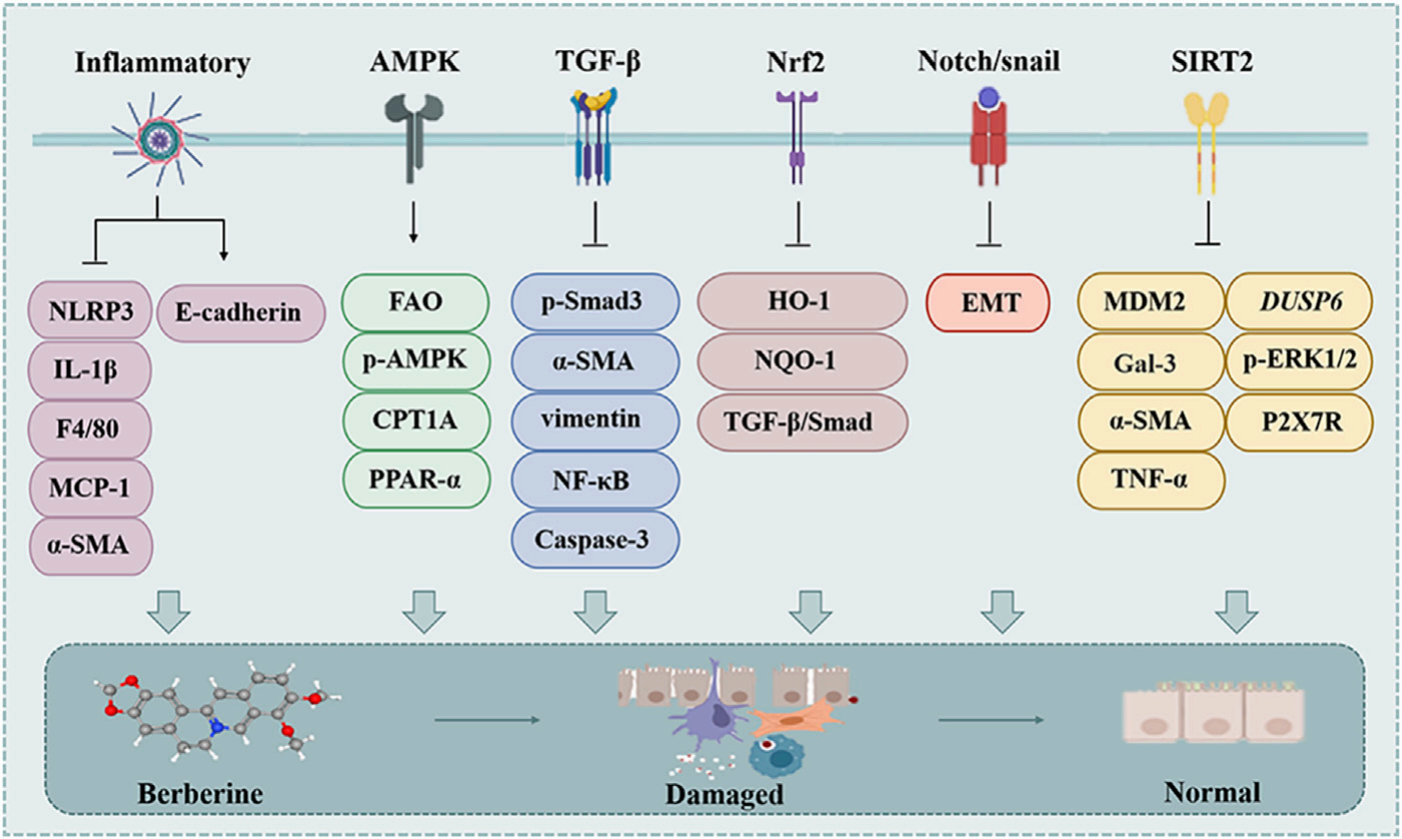
The effect of BBR on the renal fibrosis. Related signaling pathways include TGF-β, inflammatory, SDF-1/CXCR4, PPAR-γ and NF-κB pathway.

Renal fibrosis (RF) is a common manifestation of various chronic kidney diseases and representative events include increased matrix production and inhibited degradation to promote intercellular matrix interactions, cyst-like cell and fibroblast activation, tubular epithelial-mesenchymal transition, MFBs activation, immune cell infiltration and apoptosis (Sun et al., 2022). Almost all cells in the kidney are associated with the fibrotic process, including fibroblasts, tubular epithelial cells, endothelial cells, lymphocytes and macrophages (Sun et al., 2022). Thus, BBR may achieve renal protection through these cellular interactions and associated factors (Figure 5).

#### 2.4.1 TGF-β pathway

TGF-β1 is a major driver of RF, and is thought to act either directly or indirectly on a variety of cell types of the kidney to promote the fibrotic process (Meng et al., 2016).

BBR intervention can downregulate TGF-β, p-Smad3, α-SMA, vimentin, NF-κB mRNA and protein levels, relieves renal injury and inhibits RF *in vivo* and *in vitro* (Hassanein et al., 2022; Li and Zhang, 2017; Wang et al., 2014). BBR alleviates adriamycin (DOX)-induced RF through reduced TGF-β, caspase-3 and NF-κB expression and collagen deposition *in vivo*, ultimately slowing down the progression of RF (Ibrahim Fouad and Ahmed, 2021; Song et al., 2024).

#### 2.4.2 Inflammatory

RF pathogenesis mainly includes inflammation-induced tubular epithelial cell (TEC) injury, and inhibition of inflammatory progression can delay or reverse RF (Song et al., 2024; Tan E. et al., 2023). BBR (30 μM and 50 mg/kg) attenuated the extent of interstitial fibrosis and histopathological damage through inhibiting the activation of NLRP3 inflammasome and IL-1β level, which reduced the expression of F4/80, MCP-1, CoI, CoIV, and α-SMA, upregulated the level of E-cadherin, and alleviated the abnormalities of serum creatinine and urea nitrogen levels *in vivo* and *in vitro* (Song et al., 2024; Tan E. et al., 2023). In summary, BBR has a protective effect on RF.

#### 2.4.3 AMPK pathway

AMPK is a key factor contributing to the onset and progression of renal interstitial fibrosis. It can enhance mitochondrial FAO in response to decreased ATP levels (Li et al., 2022). BBR (30 μM and 50 mg/kg) intervention reversed the downregulation of p-AMPK in the kidney and reversed the downregulation of FAO-associated proteins *in vivo* and *in vitro*, including CPT1A and PPAR-α (Tan E. et al., 2023).

#### 2.4.4 Nrf2 pathway

On the one hand, BBR (200 mg/kg) was found to attenuate RF induced by STZ in C57BL/6J mice by activating the Nrf2 signaling pathway in Diabetic Nephropathy (DN); on the other hand, knockdown of Nrf2 not only counteracts BBR-induced HO-1 and NQO-1 expression, but also reverses the inhibitory effect of BBR on high glucose (HG)-induced TGF-β/Smad signaling activation and anti-fibrotic effect (Hassanein et al., 2022; Zhang X. et al., 2016).

#### 2.4.5 Notch/snail pathway

The Notch pathway has been shown to mediate cellular fibrosis such as EMT in epithelial cells in DN and is associated with TGF-β1 (Yang et al., 2017). Snail1 expression is directly regulated by the Notch signaling, and the notch/snail pathway is an important mechanism in renal interstitial fibrosis in DN (Yang et al., 2017).

BBR (30 μM and 150 mg/kg) blocked HG-induced EMT events, and inhibited expression of notch and snail1 in renal tubular epithelial cells, suppressing tubular EMT and renal interstitial fibrosis *in vivo* and *in vitro* (Yang et al., 2017).

#### 2.4.6 Sirtuin 2/murine double minute2 pathway

Class III histone deacetylase Sirtuin 2 (SIRT2) is a vital member of nicotinamide adenine dinucleotide-dependent protein deacetylases, which occupy a key position in inflammation and fibrogenesis (Ahmedy et al., 2022). BBR (200 mg/kg) inhibited cisplatin-induced SIRT2 and Murine double minute2 (MDM2) expression, further reduced hemagglutinin-3 (Gal-3), α-SMA, TNF-α, DUSP6, and reduced the mRNA levels of P2X7R and p-ERK1/2 to achieve relief of RF induced by cisplatin in female Wistar rats (Ahmedy et al., 2022).

During the development of RF, fibrotic tissue replaces nephrons in the kidneys, and the problem of increased tissue stiffness brought about by excessive accumulation of ECM hinders drug distribution and efficacy. Although various studies have shown that BBR has multiple targets and mechanisms for targeting RF (Figure 5), the above problems cannot be ignored and more researchers are called to consider them in future studies.

### 2.5 Pancreatic fibrosis

Pancreatic fibrosis is primarily associated with the activation of pancreatic stellate cells (PSCs), which results in the secretion of excess ECM proteins (An et al., 2023; Masamune et al., 2009). Pro-fibrotic mediators activate quiescent PSCs into MFBs, such as TGF-β (Masamune et al., 2009). AMPK is a metabolite sensor protein that is predominantly expressed in all organs and ameliorates inflammation and fibrosis by regulating macrophage polarization.

In the cerulein-induced pancreatic fibrosis in male Swiss albino mice, BBR dose-dependently decreased levels of pancreatic MDA, nitrite, TNF-α, IL-6, IL-1β, TGF-β1, α-SMA, COL1A, COL3A, and p-Smad2/3, reduced inflammatory cell infiltration, vesicular cell atrophy, exocrine pancreatic vacuolization, ECM deposition and EMT program; increased the content of GSH, and the expression of E-cadherin, p-AMPKα, p-AMPKβ, ACC and Smad7 expression (Bansod et al., 2020). Taken together, BBR prevented the progression of chronic pancreatitis and associated fibrosis in an AMPK-dependent manner by inhibiting TGF-β/Smad signaling and regulating macrophage polarization (Bansod et al., 2020).

### 2.6 Adipose tissue fibrosis

In the presence of overnutrition, adipose tissue undergoes rapid dynamic remodeling through adipocyte hypertrophy and hyperplasia (Koenen et al., 2021; Sun et al., 2011), accompanied by elevated accumulation of immune cells and overproduction of ECM, leading to the development of fibrosis (Li et al., 2022; Wang et al., 2018). Adipose tissue fibrosis is a hallmark of obesity-related adipose tissue dysfunction (Hu et al., 2018).

#### 2.6.1 AMPK pathway

Previous studies have shown that TGF-β signaling is associated with adipose ECM remodeling and that inhibition of TGF-β by activated AMPK alleviates adipose tissue fibrosis (Wang et al., 2018; Xu X. et al., 2021). Besides, BBR inhibits TGF-β1/Smad3 in HFD-induced white adipose tissues by attenuating macrophage infiltration and polarization, and by activating the AMPK pathway. TGF-β1/Smad3 signaling and ameliorated obesity-associated adipose tissue fibrosis (Wang et al., 2018; Xu X. et al., 2021). Notably, the inhibitory effect of BBR on adipose tissue fibrosis was blocked by compound C (an AMPK inhibitor) (Wang et al., 2018; Xu X. et al., 2021). The C57BL/6 mice adipose tissue fibrosis model used in the above study was induced by the HFD. The above results provide sufficient evidence that BBR attenuates adipose tissue fibrosis through AMPK pathway.

#### 2.6.2 Hypoxia-inducible factor 1α pathway

Hypoxia is an early event in adipose tissue dysfunction, and hypoxic conditions promote the expression of hypoxia-inducible factor 1α (HIF-1α). HIF-1α-induced transcriptional program that leads to the enhanced synthesis of ECM components and ultimately promotes the development of fibrosis in white adipose tissue (Hu et al., 2018). BBR attenuated HFD-induced fibrosis and fibroblast proliferation through reducing CoI, α-SMA, platelet-derived growth factor receptor α (PDGFR-α) and HIF-1α expression, and inhibiting aberrant ECM protein synthesis *in vivo*. All of the above results were achieved by reversing HFD-induced HIF-1α activation and transcription by BBR (Hu et al., 2018).

#### 2.6.3 Another pathway

Adipose tissue macrophages are found in up to 50% of adipose tissue in obese rodents and humans. Under obesity, the M2 macrophage phenotype is transformed into M1 macrophage phenotype. BBR regulates macrophage infiltration and polarization by decreasing the expression of iNOS, COX-2, IL-1β, IFN-γ, F4/80, MCP-1, and MMP-1α, reducing abnormal ECM deposition to reduce inflammation and fibrosis in adipose tissue through down-regulating the expression of COI, α-SMA, α-SMA, MMP-9 and TIMP-1 *in vivo* (Li et al., 2022).

### 2.7 Epidural fibrosis

Epidural fibrosis (EF) is the result of a physiological cyclic defense response during wound healing, a cycle in which fibroblasts in the healing area proliferate rapidly in response to inflammatory mediators and growth factors, and an excessive healing response leads to increased formation of scar tissue, resulting in excessive and disorganized matrix deposition (Keskin et al., 2022).

During wound healing, immune cells (such as monocytes and macrophages) together with fibroblasts and smooth muscle cells produce high levels of TGF-β1, further causes proliferation and accumulation of fibroblasts in the ECM, leading to a vicious cycle of EF after laminectomy (Gorgulu et al., 2004). In the Laminectomy-induced EF in Wistar albino rats, it's worth noting that BBR downregulates the expression of TGF-β1 further attenuate EF (Gorgulu et al., 2004). It has also been shown that BBR prevents apoptosis in human-derived myeloid cells by reducing oxidative stress induced by autophagy and endoplasmic reticulum stress induced by Ca^2+^ dysregulation (Luo et al., 2019). Other study found that BBR reduced HYP expression stopping the development of EF (Keskin et al., 2022).

Briefly, BBR plays a unique role in pancreatic, adipose tissue, and epidural fibrosis using different mechanisms, but relevant studies are insufficient to illustrate the therapeutic role of BBR in various types of organ fibrosis. Therefore, studies on the anti-fibrotic effects of BBR are indispensable in the future.

## 3 Anti-fibrotic effects of BBR derivatives

The absolute bioavailability of BBR is low and more than half of the original BBR is not absorbed by the intestinal tract, however, BBR is converted by the intestinal flora into absorbable metabolites such as compounds like dihydroberberberine (dhBBR), oxyberberine (OBB), canadine, and others (Chen et al., 2011; Feng et al., 2015; Liu et al., 2010). Previous studies have demonstrated that dhBBR reduces the release of caspase-1, apoptosis-associated speck-like protein (ASC), and IL-1β through NLRP3 inflammatory vesicle-associated mechanisms to inhibit pyroptosis (a form of programmed cell death that occurs in HF) (de Carvalho Ribeiro and Szabo, 2022; Xu L. et al., 2021). OBB treatment has been reported to increase SOD, catalase (CAT), and GPx activities, and decrease ROS, MDA, and MPO concentrations, thereby reducing oxidative stress (Dou et al., 2021). Therefore, OBB-mediated recovery of liver function may impede the progression of liver disease and promote liver regeneration (Dou et al., 2021). OBB has also been clarified to ameliorate pathological deterioration of adipocytes and hepatocytes *via* the AMPK pathway and to stimulate energy expenditure to control lipid homeostasis at a smaller dose than BBR. In addition, OBB was shown to inhibit macrophage migration and promote phenotypic conversion of M1 macrophages to M2 macrophages, ultimately reducing the inflammatory burden of the liver (Li Q. P. et al., 2021).

BBR hydrochloride (BH), which is another BBR derivative, ameliorated PM2.5-induced PF by inhibiting oxidative stress and inflammation (Zhao et al., 2023). BH concentration-dependently decreased the expression of TGF-β1, CTGF, ICAM-1, IL-1β, and p-P38, exerting anti-inflammatory and anti-fibrotic effects (Zhao et al., 2023). Postoperative adhesions are a common cause of peritoneal fibrosis. Studies have delayed that in the Surgery-induced peritoneal fibrosis in male Sprague-Dawley rats, BH reduces the expression levels of IL-1β, IL-6, TGF-β, TNF-α, ICAM-1, p-JNK and p-NF-κB, prevents adhesions after abdominal surgery, and reduces inflammation and prevents peritoneal fibrosis through inhibiting TAK1/JNK and TAK1/NF-κB signaling (Zhang Y. et al., 2014).

Additionally, it has been reported that proto-berberine alkaloids attenuate skin fibrosis by modulating mitochondrial dehydrogenase activity, cell proliferation, collagen production, and the ability of inflammatory cytokines (IL-1β and IL-6) production *in vitro* (Pietra et al., 2015).

## 4 Pharmacokinetics

BBR has a low oral bioavailability due to poor intestinal absorption and rapid metabolism, Hua et al. used the liquid chromatography-electrospray ionization-mass spectrometry method to demonstrate that plasma concentrations were only 0.4 ng/mL at an oral dose of 400 mg (Hua et al., 2007). Oral bioavailability of BBR is hampered by intestinal first-pass elimination, hepatic distribution and p-glycoprotein (Pg-P) pumps (Imenshahidi and Hosseinzadeh, 2016; Liu et al., 2010). After 4 h of oral administration of BBR (200 mg/kg) to rats, the distribution of BBR in organs was at least 10–30 times higher than that in plasma, with the highest distribution in the liver, followed by the kidney, this may provide an explanation for the pharmacologic effects of BBR in clinical human disease (Kumar et al., 2015; Tan et al., 2013; Wang et al., 2005). The metabolites of BBR are mainly berberrubine (up to 65.1%), thalifendine, demethylene-berberine and jatrorrhizine, which are ultimately excreted in urine, bile and feces (Feng X. et al., 2019).

The bioavailability of BBR is the biggest hindrance to its clinical application, and several strategies have been found to improve the bioavailability of BBR (Imenshahidi and Hosseinzadeh, 2016). For example, Chitosan-N-Acetylcysteine and β-cyclodextrin modulated Pg-P activity to further enhanced the intestinal absorption of BBR (Imenshahidi and Hosseinzadeh, 2016). Chen et al. found that 2.5% D-α-tocopherol polyethylene glycol 1,000 succinate increased the area under the curve (AUC) (0–36) of BBR up to 1.9-fold, possibly through inhibition of Pg-P activity (Chen et al., 2011). Godugu et al. prepared a mucoadhesive microparticle formulation of BBR that enhanced the AUC of BBR up to 6.98-fold (Godugu et al., 2014), whereas oral BBR microemulsion formulation increased the bioavailability of BBR up to 6.47-fold (Gui et al., 2008). Wang et al. improved the oral bioavailability of BBR up to 2.4-fold by an anhydrous reverse micelle delivery system (Wang et al., 2011). Current clinical studies could not adequately address the issue of BBR bioavailability, and there is still a need to construct more drug formulations to address this challenge in the future, so as to increase the utilization and the application range of BBR.

## 5 Safety and toxicology

In general, BBR is virtually safe at routine doses, with low toxicity and side effects (Imenshahidi and Hosseinzadeh, 2016; Liu et al., 2016), such as only mild gastrointestinal reactions (diarrhea and constipation) reported in clinical studies (Zhang et al., 2010). On the one hand, BBR may prevent toxic reactions and side effects associated with some anti-tumor and analgesic drugs, such as cisplatin, cyclophosphamide, bleomycin, and acetaminophen (Chitra et al., 2013; Domitrovic et al., 2013a; Germoush and Mahmoud, 2014). On the other hand, in some cases, other adverse effects may occur. 10 mg/kg of BBR can suppress immune function in mice (Mahmoudi et al., 2016), and BBR interaction with macrolides and statins may lead to cardiac arrhythmias and reduce the efficacy of the drug (Feng P. et al., 2018; Holy et al., 2009; Zhi et al., 2015). Therefore, we conclude that BBR is safe for use in oral formulations based on traditional dosages and indications.

## 6 Discussion

Although a small number of drugs are available for the treatment of fibrosis, such as Nidanib and Pirfenidone, long drug cycles, adverse effects, and the lack of drugs targeting different organs remain a challenge to clinical treatment. Therefore, effective therapeutic strategies for fibrosis are urgently required. Natural products have become a hotspot for research and new drug development in recent years due to their advantages of multiple actions, targets and pathways, and more and more natural products have been reported with the utilization of various biological techniques. Studies over the past 2 decades have convincingly demonstrated the therapeutic effects of BBR on various types of fibrosis. The efficacy of BBR in the treatment of different types of fibrosis is mediated by its multi-target pharmacological profile, including the modulation of the TGF-β/Smad, inflammation, AMPK, and Nrf2 pathways, among others (Table 1). Although numerous studies have described the antifibrotic effects of BBR, most of them are based on cellular and animal studies, and comprehensive clinical studies are still lacking.

**TABLE 1:** Anti-fibrotic effects of BBR.

Disease	*In vivo*/*vitro*	Animal/cell model	Dosage	Duration	Mode of administration	Described effects	Pathways	Refers
Hepatic fibrosis	*In vivo*	Db/db male mice	200 mg/kg	5 weeks	Gavage	↓: α-SMA, CoI, ALT, AST, PDGF, CTGF, MMP-1, SREBP-1c, CHREBP, FAS, C/EBPβ, ROS, CYP2E1, ATF6, XBP1, ATF4, CHOP, TNF-α, IL-6, α-SMA, TIMP-1, TGF-β1	Endoplasmic reticulum stress; Metabolism	Zhang et al. (2016b)
	*In vivo*	Tunicamycin-induced C57BL/6 mice	75, 150, 300 mg/kg	72 h	Gavage	↓: *CHOP*, *Grp78, ATF6*, SCD1	Endoplasmic reticulum stress; Metabolism	Yang et al. (2022)
	*In vivo*	CCl4-induced ICR mice	25, 50 mg/kg	6 weeks	Gavage	↑: p-AMPK/AMPK, SOD ↓: p-Akt/Akt, MDA, NOX4, ROS	AMPK	Li et al. (2014)
	*In vivo*	CCl4-induced male mice	200 mg/kg	6 weeks	Gavage	↑: Ptgs2, ROS ↓: HA, ALT, AST, p75^NTR^, α-SMA, LC3B, FTH1	Ferroptosis	Yi et al. (2021)
	*In vivo*	CCl4-induced male Balb/c mice CCl4-induced Male Wistar rats CCl4-induced male KM mice	3, 9 mg/kg 50, 100, 200 mg/kg 120 mg/kg	2 weeks 4 weeks 7 weeks	Gavage	↑: MMP-2, SOD ↓: TNF-α, α-SMA, TGF-β1, MMP-9, ALT, AST, MDA, HYP	Oxidative Stress	Domitrovic et al. (2013b), Zhang et al. (2008)
	*In vitro*	HepG2; LX-2; THP-1	5 µM	72 h	—	↓: ROS, MIP-1α, MIP1β, MCP-1, IL-7, CoI	Oxidative Stress	Rafiei et al. (2023)
	*In vivo*	HFD-induced male SpragueDawley rats	100 mg/kg	16 weeks	Gavage	↑: SIRT3, p-AMPK, p-ACC, CPT-1A, HDL-C ↓: TC, TG, LDL-C, ALT, AST	Metabolism	Zhang et al. (2019)
	*In vivo*	LPS-induced male Sprague–Dawley rats	100 mg/kg	0, 24, 48 h	Gavage	↓: COX-2, p-p38, p-ATF2, ATF2, ATF3	Metabolism	Feng et al. (2012)
	*In vitro*	LPS-stimulated PBMC	25, 50, 100 µM	6, 12, 24 h	—	↓: COX-2	Metabolism	Guo et al. (2008)
	*In vivo*	Streptozotocin-induced rats	75, 150, 300 mg/kg	16 weeks	Gavage	↑: HDL-C, Apo-AI, PPAR-α ↓: TC, TG, LDL-c, ApoB, PPAR-γ	Metabolism/PPARs	Zhou et al. (2008)
	*In vivo*	HFD-induced male Wistar rats	100 mg/kg	18 weeks	Gavage	↓: LBP, MCP-1	Gut Microbiota	Zhang et al. (2012)
	*In vitro* *In vivo*	CFCS CCl4-induced male C57BL/6 mice	5, 10, 20 μg/mL 100, 200, 400 mg/kg	48 h 6 weeks	Gavage	↑: p21, p27 ↓: p-FoxO1 (Ser-256), p-Akt	FoxO	Sun et al. (2009)
	*In vitro* *In vivo*	HSCs; LX-2 CCl4-induced male C57BL/6 mice	0, 1, 5, 10, 20 µM 50 mg/kg	0, 3, 6, 12, 24 h 4 weeks	Gavage	↑: miR-30a-5p, p53, BAX, cleaved PARP, p62 ↓: ATG5, BCL-2, HYP, α-SMA, 1-A1, LC-3, LC3-II	miR-30a-5p /ATG5/Autophagy	Tan et al. (2023b)
Myocardial fibrosis	*In vivo*	ISO-induced male SD rats	10, 30, 60 mg/kg	10 days	Gavage	↓: TGF-β1, COL1A1, COL3A1, CTGF, α-SMA	TGF-β/Smad	Che et al. (2019)
	*In vivo* *In vitro*	H9c2; Ang II-induced cardiac fibroblasts TAC-induced male SD rats	0, 1, 10 µM 10 mg/kg	6 weeks	Gavage	↑: IL-10, p-AMPK ↓: p-mTOR, p-4EBP1, p-p70S6K (Thr389), CoI, CoIII, TGF-β1, MMP-2, MMP-9	AMPK	Chen et al. (2020)
	*In vivo*	DOX-induced male Sprague-Dawley rats	60 mg/kg	3 weeks	Gavage	↑: SOD, ROS, MDA ↓: α-SMA, CoI, CoIII, MDA	Nrf2	Wang et al. (2023)
Pulmonary fibrosis	*In vivo*	BLM-induced male Wistar albino rats	100, 150, 200, 250, 300 mg/kg	1, 2, 3 weeks	Gavage	↑: Samd7, PTEN, Beclin-1, LC3-II ↓: p-Smad2, α-SMA, CoI, CoIII, p-FAK, p-PI3K, p-Akt, p-mTOR	TGF-β/Smad	Chitra et al. (2013)
	*In vivo*	BLM-induced male Wistar albino rats	200 mg/kg	2 weeks	Gavage	↓: HYP, CXCL14, CXCR4, α-SMA, CoI, CoIII, MMP-2, MMP-9, p-Smad2/3	TGF-β/Smad	Li et al. (2019)
	*In vivo*	PM2.5-induced male C57BL/6 mice	50 mg/kg	45 days	Gavage	↑: E-cadherin, PPAR-γ ↓: HYP, LDH, MDA, IL-6, IL-1β, TNF-α, caspase3, caspase9, FN, COI, COIII, α-SMA	Inflammatory/PPAR-γ	Zhao et al. (2023)
	*In vivo*	BLM-induecd male Kunming mice	50 mg/kg	2, 6 weeks	Gavage	↓: TNF-α, IL-6, IL-8, TGF-β1, PDGF-AB, HYP, α-SMA, p38MAPKα, p38MAPKα (pT180/Y182)	Inflammatory	Yang et al. (2023)
	*In vivo*	BLM-induecd albino male mice	5 mg/kg	2 weeks	Gavage	↑: Nrf2 ↓: SDF-1, CXCR4, TGF-β, p-Smad2/3, α-SMA, NF-κB p65, TNF-α, IL-6, MDA, GSH, SOD, CAT	SDF-1/CXCR4	Ahmedy et al. (2023)
	*In vivo*	BLM-induecd female ICR mice	50, 100, 200 mg/kg	3 weeks	Gavage	↑: HGF, PTEN, PPAR-γ, CD36, AP2 ↓: HYP	PPAR-γ	Guan et al. (2018)
	*In vivo*	BLM-induecd male wistar albino rats	200 mg/kg	4 weeks	Intravenous injection	↑: Nrf2, SOD, CAT, GPx, GR ↓: MDA, OH, NO, MPO, IκB, NF-κB p65, iNOS, TNF-α, TGF-β1	NF-κB	Chitra et al. (2013)
Renal fibrosis	*In vivo* *In vitro*	TGF-β1-stimulated HK-2 UUO-induced male C57BL/6 mice	30 µM 50 mg/kg	24 h 2 weeks	Gavage	↑: p-AMPK, CRT1A, PPAR-α, E-cadherin, FAO ↓: F4/80, MCP-1, NLRP3, IL-1β, Bcl-2, Bax, caspase-3, α-SMA	TGF-β/Inflammatory /AMPK	Tan et al. (2023a)
	*In vivo*	UUO-induced male Sprague–Dawley rats	200 mg/kg	2 weeks	Gavage	↑: SOD, CAT ↓: MDA, ED-1, MPO, TGF-β1, p-Smad3, α-SMA	TGF-β	Wang et al. (2014)
	*In vivo*	STZ-induced male Wistar rats	400 mg/kg	12 weeks	Gavage	↑: SOD, CAT ↓: Scr, BUN, TGF-β, α-SMA, vimentin	TGF-β	Li and Zhang, (2017)
	*In vivo*	DOX-induced male Wistar rats	50 mg/kg	2 weeks	Gavage	↑: SOD, CAT ↓: MDA, H_2_O_2_, TGF-β1, caspase-3, NF-κBp65	TGF-β	Ibrahim Fouad and Ahmed, (2021)
	*In vivo*	STZ-induced C57BL/6J mice	200 mg/kg	3 weeks	Gavage	↑: Nrf2, NQO1, HO-1 ↓: α-SMA, CoI, E-cadherin, p-Smad2/3	Nrf2	Zhang et al. (2016a)
	*In vivo* *In vitro*	mRTECs Female KKAy mice	30 µM 150 mg/kg	40 min 4 weeks	Gavage	↑: Nrf2, NQO1, HO-1, E-cadherin ↓: Cr, BUN, α-SMA, snail, jagged1, notch1, hes1	Notch/snail	Yang et al. (2017)
	*In vivo*	Cisplatin-induced female Wistar rats	200 mg/kg	2 weeks	Gavage	↑: Nrf2, NQO1, HO-1, E-cadherin ↓: BUN, KIM-1, Gal-3, α-SMA, TNF-α, P2X7R, p-ERK1/2, DUSP6, SIRT2, MDM2	SIRT2/MDM2	Ahmedy et al. (2022)
Pancreatic fibrosis	*In vivo*	Cerulein-induced male Swiss albino mice	3, 10 mg/kg	3 weeks	Intravenous injection	↑: GSH, p-AMPKα, p-AMPKβ ↓: MDA, TNF-α, IL-6, IL-1β, TGF-β1, α-SMA, COI1A, COI3A, p-Smad2/3, CD206	AMPK	Bansod et al. (2020)
Adipose tissue fibrosis	*In vivo*	HFD-induced male C57BL/6 mice	75, 150 mg/kg	4 weeks	Gavage	↓: COI1A1, COI3A1, CoI6A3, MMP-2, MMP-9, TIMP-1, LOX, TGF-β1, p-Smad3, TNF-α, iNOS	AMPK	Wang et al. (2018)
	*In vivo*	HFD-induced male C57BL/6 mice	100, 200, 300 mg/kg	8 weeks	Gavage	↓: COI, α-SMA, PDGFR-α, HIF-1α	HIF-1α	Hu et al. (2018)
	*In vivo*	HFD-induced male C57BL/6J mice	200 mg/kg	20 weeks	Gavage	↑: SIRT3 ↓: TC, TG, LDL-C, NEFA, iNOS, COX-2, IL-1β, IFN-γ, F4/80, MCP-1, MMP-1α, Ccl5, Ccl11, Cx3cl1, Cxcl10, COI, α-SMA, PDGFR-α, HIF-1α, α-SMA, MMP-9, TIMP-1, p-ERK, p-JNK, p-p38, p-IKKα/β, p-IκBα, p-p65	SIRT3/Inflammatory	Li et al. (2022)
Epidural fibrosis	*In vivo*	Laminectomy-induced Wistar albino rats	10, 60 mg/kg	1 week	Gavage	↓: HYP	HYP	Keskin et al. (2022)
Peritoneal fibrosis	*In vivo*	Surgery-induced male Sprague-Dawley rats	0.75, 1.5 mg/mL	2 weeks	Dropped into the abdominal cavity	↓: IL-1β, IL-6, TGF-β, TNF-α, ICAM-1, p-JNK, p-NF-κB, TAK1	Inflammatory	Zhang et al. (2014b)
Skin fibrosis	*In vitro*	H_2_O_2/_TGF-β1-stimulated HDF	1–10 µM	18, 42 h	—	↓: TGF-β1, IL-1β, IL-6	Inflammatory	Pietra et al. (2015)

As mentioned earlier, the low bioavailability of BBR is the biggest hindrance to its practical application, and its preparation into modern oral dosage forms is essential, such as a mucoadhesive microparticle formulation and microemulsion formulation, as it reduces side effects and achieves better therapeutic effect (Feng X. et al., 2019). Besides, the development and advancement of various biological technologies can also solve this problem, such as an anhydrous reverse micelle delivery system, polymer materials and nanotechnology. Generally, BBR at routine doses is almost safe with low toxicity and side effects, the adverse effects of BBR under special circumstances may also limit the clinical application of BBR, so it needs to be used according to conventional dosage and indications.

Interestingly, despite the poor bioavailability of BBR, this does not explain why it is so effective *in vivo*. Growing research suggests that BBR may target the gut microbiota as a target for multifunctional action, exerting therapeutic effects by reversing the structure and value of the gut microbiota under pathological conditions (Feng X. et al., 2019). A study on beagle dogs showed that continuous oral administration of BBR for 7 days upregulated the levels of butyrate (a product of bacteria) and nitroreductases-producing bacteria in the plasma (Feng R. et al., 2018). Wang et al. (2017) found different bioavailability of BBR in obese and lean animals due to different structural distribution and values of microbiota. Alioga et al. (2016) studied the differential expression of gut microbiota between populations from different countries, emphasizing that the gut microbiota is closely related to inter-individual and inter-ethnic differences in drug metabolism. BBR is rapidly metabolized *in vivo*, and the gut microbiota enhances the bioactivity of BBR by converting it to dhBBR. Nevertheless, the effects of dhBBR treatment given alone differed from those described above, suggesting that the anti-fibrotic effect of BBR is not only due to its metabolites. Another study confirmed that the intestinal absorption of dhBBR in the body is 5-fold higher than that of BBR, and the absorbed dhBBR is oxidized to BBR and re-enters the bloodstream (Feng et al., 2015). There is also a study that explains this, where BBR undergoes extrahepatic metabolism and intestinal conversion to OBB, which are mainly transported intracellularly in a protein-bound form to form hepatocyte-targeted and enterohepatic circulations (Huang et al., 2023). We believe that the distribution of BBR and its bioactive metabolites *in vivo* may partially explain this.

## 7 Final considerations and prospect

Natural products are derivatives of traditional Chinese medicines and are the material basis for the pharmacological effects. They have the advantages of multiple actions, multiple targets and multiple pathways, and have become a hot spot for research and new drug development in recent years. BBR has attracted much attention due to its multiple pharmacological effects. Numerous studies on BBR affecting various types of fibrosis have found that its mechanisms of action include, but are not limited to the regulation of collagen homeostasis, anti-inflammation, anti-oxidative stress, and the prevention of tissue damage through different signaling cascades, and the mechanisms of action vary in different tissues or organs. We are only reviewing the completed studies with some limitations. Firstly, the bioavailability of BBR is insufficient, and the discovery of good drug carriers is important for improving its solubility, enhancing tissue targeting, and expanding the range of clinical applications. Secondly, further comprehensive studies, especially clinical trials, are urgently needed, which are essential for elucidating other mechanisms and molecular targets of BBR in the treatment of fibrosis as well as evaluating its efficacy and safety.

## Figures and Tables

**Table:** 

**Abbreviations**	
**BBR**	Berberine
**ECM**	extracellular matrix
**TGF-β**	transforming growth factor-β
**IPF**	idiopathic pulmonary fibrosis
**MFBs**	myofibroblasts
**CoI**	Collagen I
**CoIII**	Collagen III
**HF**	hepatic fibrosis
**HSCs**	hepatic stellate cells
**ROS**	reactive oxygen species
**ERS**	endoplasmic reticulum stress
**α-SMA**	smooth muscle α-actinin
**ALT**	alanine aminotransferase
**AST**	azelaic transaminase
**PDGF**	platelet-derived growth factor
**CTGF**	connective tissue growth factor
**MMP-1**	metalloproteinase-1
**AMPK**	AMP-activated protein kinase
**CCl4**	carbon tetrachloride
**SOD**	superoxidedismutase
**MDA**	Malondialdehyde
**NOX4**	NADPH oxidase 4
**HA**	hyaluronic acid
**TNF-α**	Tumor necrosis factor-α
**HYP**	hydroxyproline
**PPARs**	Peroxisome proliferator-activated receptors
**SCFAs**	Short-chain fatty acids
**MCP-1**	monocyte chemotactic protein-1
**FoxO**	Forkhead box O
**Akt**	protein kinase B
**CFSCs**	hepatic stellate cells
**miR**	microRNAs
**COL1A1**	collagen type I alpha
**COX-2**	Cyclooxygenase-2
**MF**	myocardial fibrosis
**COL3A1**	collagen type III alpha
**Nrf2**	Nuclear factor-erythroid 2-related factor 2
**GPx**	Glutathione peroxidase
**PF**	Pulmonary fibrosis
**EMT**	epithelial-mesenchymal transition
**BLM**	bleomycin
**SDF-1**	Stromal cell-derived factor-1
**RF**	Renal fibrosis
**DN**	Diabetic Nephropathy
**SIRT2**	Sirtuin 2
**MDM2**	Murine double minute2
**NF-κB**	nuclear factor-κB
**Gal-3**	hemagglutinin-3
**PSCs**	pancreatic stellate cells
**HIF-1α**	hypoxia-inducible factor 1α
**EF**	Epidural fibrosis
**dhBBR**	dihydroberberberine
**OBB**	oxyberine
**BH**	BBR hydrochloride
